# Comparison of CPR quality and rescuer fatigue between standard 30:2 CPR and chest compression-only CPR: a randomized crossover manikin trial

**DOI:** 10.1186/s13049-014-0059-x

**Published:** 2014-10-28

**Authors:** Jonghwan Shin, Seong Youn Hwang, Hui Jai Lee, Chang Je Park, Yong Joon Kim, Yeong Ju Son, Ji Seon Seo, Jin Joo Kim, Jung Eun Lee, In Mo Lee, Bong Yeun Koh, Sung Gi Hong

**Affiliations:** Department of Emergency Medicine, Seoul Metropolitan Government-Seoul National University Boramae Medical Center, Seoul, South Korea; Department of Emergency Medicine, Sungkyunkwan University School of Medicine, Samsung Changwon Hospital, Changwon, South Korea; Department of Emergency Medicine, Gachon University Gill Hospital, Incheon, South Korea; Depatment of Emergency Medical Technology, Dongnam Health University, Suwon, South Korea

**Keywords:** Cardiopulmonary resuscitation, Fatigue, Chest compression, Heart rate

## Abstract

**Objective:**

We aimed to compare rescuer fatigue and cardiopulmonary resuscitation (CPR) quality between standard 30:2 CPR (ST-CPR) and chest compression only CPR (CO-CPR) performed for 8 minutes on a realistic manikin by following the 2010 CPR guidelines.

**Methods:**

All 36 volunteers (laypersons; 18 men and 18 women) were randomized to ST-CPR or CO-CPR at first, and then each CPR technique was performed for 8 minutes with a 3-hour rest interval. We measured the mean blood pressure (MBP) of the volunteers before and after performing each CPR technique, and continuously monitored the heart rate (HR) of the volunteers during each CPR technique using the MRx monitor. CPR quality measures included the depth of chest compression (CC) and the number of adequate CCs per minute.

**Results:**

The adequate CC rate significantly differed between the 2 groups after 2 minutes, with it being higher in the ST-CPR group than in the CO-CPR group. Additionally, the adequate CC rate significantly differed between the 2 groups during 8 minutes for male volunteers (p =0.012). The number of adequate CCs was higher in the ST-CPR group than in the CO-CPR group after 3 minutes (p =0.001). The change in MBP before and after performing CPR did not differ between the 2 groups. However, the change in HR during 8 minutes of CPR was higher in the CO-CPR group than in the ST-CPR group (p =0.007).

**Conclusions:**

The rate and number of adequate CCs were significantly lower with the CO-CPR than with the ST-CPR after 2 and 6 minutes, respectively, and performer fatigue was higher with the CO-CPR than with the ST-CPR during 8 minutes of CPR.

## Background

According to the 2010 cardiopulmonary resuscitation (CPR) guidelines, all rescuers should provide chest compressions (CC) to victims of cardiac arrest. In addition, high quality chest compression, which is defined as a compression depth of at least 5 cm and a rate of at least 100 CCs per minute, should be performed by a lay rescuer or healthcare provider. This recommendation emphasizes deeper and faster CCs than previous CPR guidelines. Therefore, the lay rescuer or healthcare provider may become exhausted more quickly during CPR. Rescuer fatigue may lead to inadequate CC rates and/or depth [1,2]. Significant fatigue and shallow CCs are common after 1 minute of CPR, although rescuers may not recognize that fatigue is present for 5 minutes [2]. When 2 or more rescuers are available, they can take turns in performing CC by switching approximately every 2 minutes to prevent decreases in the quality of CC. However, only 1 rescuer is available for many cardiac arrest cases, specifically in the home setting. In such cases, 1 rescuer must perform CPR alone for a few minutes or more until the emergency medical technicians (EMT) or healthcare provider arrives at the scene. The 2010 CPR guidelines encourage CC-only CPR (CO-CPR) for the untrained lay rescuer owing to reluctance to perform mouth-to-mouth ventilation for unknown victims of cardiac arrest, and because it is a simplified method of CPR. Observational studies of adults with cardiac arrest treated by lay rescuers have shown similar survival rates among victims receiving CO-CPR versus standard 30:2 CPR (ST-CPR) with rescue breaths [3-7]. CO-CPR helps victims after sudden cardiac arrest with ventricular fibrillation because the oxygen level in the blood remains adequate for the first several minutes [8-10]. CO-CPR is not as effective as ST-CPR for cardiac arrests of non-cardiac origin in adults and children, and when prolonged CPR is needed [11,12]. CCs combined with rescue breaths are therefore the method of choice for CPR delivered by both trained lay rescuers and professionals. There is no clinical study on the comparison of single rescuer fatigue and CPR performance between ST-CPR and CO-CPR according to the 2010 CPR guidelines. In this study, we aimed to compare rescuer fatigue and CPR quality between ST-CPR and CO-CPR performed on a realistic manikin according to the 2010 CPR guidelines.

## Methods

### Study design

This study was a prospective randomized crossover simulation trial conducted in a large urban training hospital. This design was chosen to show differences in the fatigue and performance level following 2 different CPR methods by using each subject as his or her own control. This study was approved by the Institutional Review Board of our hospital, and written informed consent was obtained from each participant.

### Study setting and population

Before the main study, we conducted a pilot study to compare the difference in CPR performance during 8 minutes of CPR. The pilot study showed a 30% difference in the adequate CC rate (more than 5 cm) during 8 minutes, ST-CPR had a higher rate of adequate CCs than CO-CPR. The results of that study indicated that a sample size of 35 subjects in each group was required to show a difference between ST-CPR and CO-CPR during 8 minutes of CPR with a power of 80% and a significance level of 0.05. First year EMT university students were recruited via a class presentation (18 men and 18 women). We explained to the participants that the study compared fatigue and performance of 2 different types of CPR techniques performed for 8 minutes. After all of the participants signed a consent form, they participated in a layperson BLS training simulation course for 3 hours.

### Study protocol

Participants were grouped by gender and then randomized to either the ST-CPR group or the CO-CPR group at first. ST-CPR was defined as 30:2 single rescuer CPR (30 CCs at a rate of 110 per minute, followed by 2 ventilations and rescuers should give each rescue breath over about 1 second) for 8 minutes, and CO-CPR was defined as CO-CPR (continuous chest compression at a rate of 110 per minute) for 8 minutes. Participants of group 1 first performed ST-CPR and then CO-CPR after a 3-hour rest. Participants of group 2 first performed CO-CPR and then ST-CPR after a 3-hour rest. Participants of groups 1 and 2 were further divided into 4 classes according to the gender and were assigned a test room number. Room 1 (male participants numbered 1 to 9 with 2 male examiners), room 2 (male participants numbered 10 to 18 with 2 male examiners), room 3 (female participants numbered 1 to 9 with 2 female examiners), and room 4 (female participants numbered 10 to 18 with 2 female examiners). Each participant performed 2 different types of CPR in the same room after a 3-hour rest. The reason for separating the groups into rooms was that electrodes for electrocardiographic monitoring were attached to the upper body of each subject by a same-gender examiner, and saving the execution time was needed. We prepared 4 simulation manikins with a portable computer and 4 electrocardiograph monitors for recording. After each participant entered his or her assigned room, the examiner first measured his or her blood pressure and attached an electrode to his or her trunk for continuous monitoring of the subject’s heart rate with an MRx monitor (HeartStart, Phillips, Netherlands). The examiners then instructed the participants to perform the appropriate CPR technique for 8 minutes. At the same time, an electronic metronome with an audible beeping tone was used to guide the chest compression rate. After each participant completed a CPR session of 8 minutes, the examiner measured his or her blood pressure.

### Outcome measures

We measured the following baseline characteristics of participants: age, gender, height (cm), weight (kg), and body mass index. In addition, we assessed the following clinical parameters of participants: blood pressure (mmHg; before and after each type of CPR) and continuous heart rate (beats per minute). We used Skill Reporter Resusci Anne (Laerdal Medical, Stavanger, Norway) for all of the participants. Compression depth, compression rate, number of CCs per minute, and change in compression depth over time were measured to assess the CPR performance. Compression depth was divided into more than 5 cm and more than 4 cm, because the analysis of compression depth more than 4 cm would be compared with that in previous studies using the 2005 CPR guidelines. In addition, recoil failure after chest compression (rate) and hands-off time (seconds per 2 rescue breaths) were recorded. No subjective fatigue scale measurement was taken. However, we asked the participants to state, “I’m exhausted”, or to stop CPR in the event that they experienced extreme fatigue during each type of CPR. No such event occurred during this study.

### Data analysis

Data were analyzed using SPSS 20.0 (SPSS Inc., Chicago, IL). Numerical data on basic characteristics and compression recoil failure were analyzed by the *t*-test. Differences in the rate and number of adequate CCs per minute between the ST-CPR and CO-CPR groups were analyzed by the paired *t*-test and generalized estimating equations for changes in total periods. A p value <0.05 was considered to be statistically significant.

## Results

**Figure 1:**
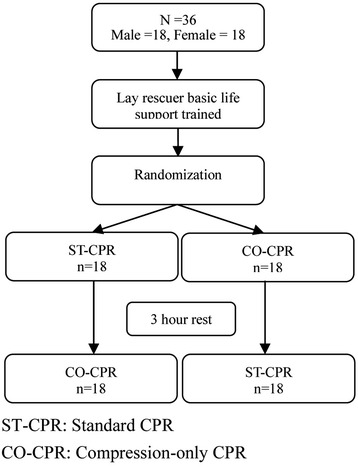
**Study protocol.**

A total of 36 first-year EMT students were enrolled in this study and they provided informed consent (Figure 1). None of the participants were excluded between the time of the layperson simulation course and the main study. Demographic data of all participants are shown in Table 1.

**Table 1:** **Demographic characteristics of participants**

	**Total**	**Males**	**Females**
Number of subjects	36	18	18
Age (years)	18.7 ± 1.0	18.6 ± 0.7	18.8 ± 1.3
Height (cm)	169.2 ± 8.1	175.6 ± 4.3	162.8 ± 5.4
Weight (kg)	61.5 ± 8.7	67.5 ± 6.3	55.4 ± 6.4
BMI	21.4 ± 1.9	21.9 ± 1.8	20.9 ± 1.9

### Comparison of the rate of adequate CCs according to depth between groups

**Figure 2:**
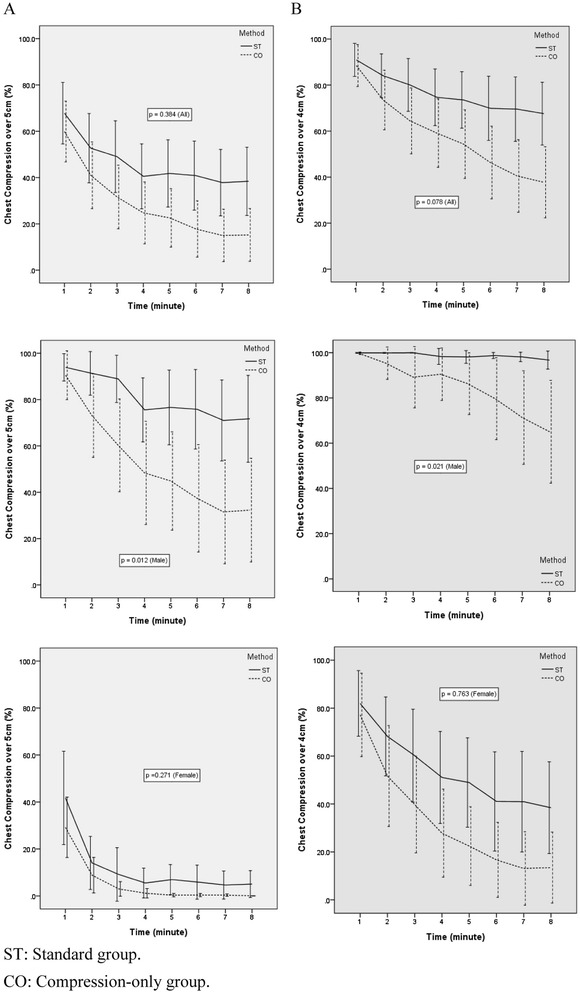
**The change in the rate and number of adequate chest compressions per minute with each CPR method. A**. Chest compression depth more than 5 cm. **B**. Chest compression depth more than 4cm.

The rate of adequate CCs per minute was not significantly different between the 2 types of CPR during the first 1 minute (p =0.136), but it was significantly different during each minute after 2 minutes (Table 2). However, the difference in the rate of adequate CCs per minute between the 2 groups during the total 8 minutes was not statistically significant (p = 0.384) (Figure 2A). When the analysis was performed on the basis of gender, the rate of adequate CCs was statistically significantly different between the 2 groups during the total 8 minutes for men (p =0.012), but not for women (p =0.271). The rate of CCs with a depth of more than 4 cm per minute was not significantly different between the 2 types of CPR during the first and second minute (p = 0.572, p =0.093, respectively), but it was significantly different after 3 minutes. However, the difference in the rate of CCs with a depth of more than 4 cm per minute between the 2 groups during the total 8 minutes was not statistically significant (p =0.078) (Figure 2B). When the analysis was performed on the basis of gender, the rate of adequate chest compressions with a depth of more than 5 cm was significantly different between the 2 groups during the total 8 minutes for male participants (p =0.021), but not for female participants (p =0.763).

**Table 2:** **The rate of chest compressions per minute according to each compression depth**

	**Rate of chest compressions per minute with a depth of more than 5 cm (mean ± SD, %)**			**Rate of chest compressions per minute with a depth of more than 4 cm (mean ± SD, %)**		
**Time (minute)**	**ST-CPR**	**CO-CPR**	**p-value**	**ST-CPR**	**CO-CPR**	**p-value**
1	68 ± 39	60 ± 39	0.136	91 ± 21	89 ± 27	0.572
2	52 ± 45	41 ± 43	0.015	84 ± 28	74 ± 38	0.093
3	49 ± 46	32 ± 41	0.003	80 ± 34	65 ± 42	0.019
4	41 ± 42	25 ± 40	0.004	75 ± 36	59 ± 44	0.009
5	42 ± 23	23 ± 37	0.001	74 ± 36	54 ± 44	0.002
6	41 ± 44	21 ± 37	0.001	70 ± 41	49 ± 46	0.002
7	38 ± 42	18 ± 35	0.001	70 ± 41	44 ± 46	<0.001
8	38 ± 44	18 ± 35	0.001	68 ± 40	41 ± 45	<0.001

### Comparison of the number of chest compressions between groups

**Figure 3:**
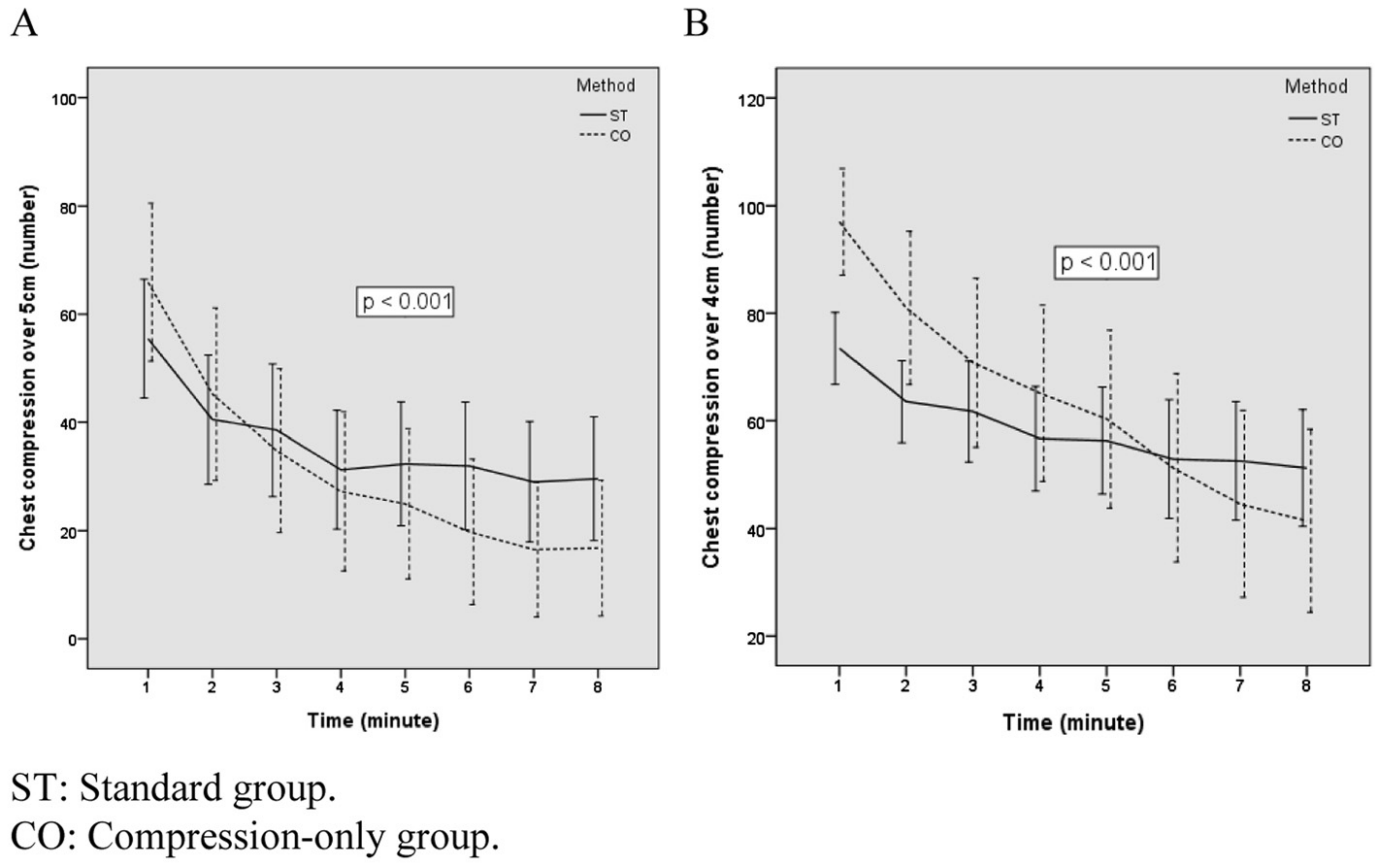
**The change in number of adequate chest compressions with each CPR method. A**. Chest compression depth more than 5 cm. **B**. Chest compression depth more than 4cm.

The total number of CCs per minute was higher in the CO-CPR group than in the ST-CPR group (p <0.001) (Table 3). This result reflects a time of 2 rescue breaths in 30:2 ST-CPR, and the mean hands-off time was 3.9 ± 4.1 seconds every 2 rescue breaths during ST-CPR. The number of adequate CCs was lower in the ST-CPR group than in the CO-CPR group during the first 2 minutes, but it was higher in the ST-CPR group after 3 minutes (Table 3, Figure 3A). Furthermore, the difference in the number of adequate compressions between the 2 groups was statistically significant during the full 8 minutes (p =0.001). The number of CCs with a depth of more than 4 cm was lower in the ST-CPR group than in the CO-CPR group during the first 5 minutes, but it was higher in the ST-CPR group after 6 minutes (Table 3, Figure 3B). In addition, the difference in the number of chest compressions with a depth of more than 4 cm between the 2 types of CPR was significant during the total 8-minute period (p =0.001).

**Table 3:** **The total number of CCs with each CPR method and the number of adequate chest compressions with each compression depth and CPR method**

	**Total number of chest compressions**			**Adequate number of chest compressions with a depth of more than 5cm**			**Adequate number of chest compressions with a depth of more than 4 cm**		
**Time (minute)**	**ST-CPR**	**CO-CPR**	**p-value**	**ST-CPR**	**CO-CPR**	**p-value**	**ST-CPR**	**CO-CPR**	**p-value**
1	80 ± 9	109 ± 6	0.001	55 ± 33	67 ± 44	0.048	73 ± 20	97 ± 29	<0.001
2	77 ± 7	110 ± 7	0.001	41 ± 35	45 ± 47	0.309	64 ± 23	81 ± 42	0.007
3	77 ± 9	110 ± 6	0.001	39 ± 36	35 ± 45	0.464	62 ± 28	71 ± 46	0.185
4	76 ± 9	110 ± 7	0.001	31 ± 32	27 ± 43	0.428	57 ± 29	65 ± 48	0.161
5	76 ± 9	110 ± 7	0.001	32 ± 34	25 ± 41	0.128	56 ± 29	60 ± 49	0.509
6	75 ± 9	111 ± 8	0.001	31 ± 35	20 ± 39	0.042	52 ± 33	51 ± 50	0.963
7	75 ± 9	111 ± 8	0.001	27 ± 32	17 ± 36	0.040	51 ± 33	45 ± 50	0.342
8	76 ± 10	111 ± 9	0.001	29 ± 34	17 ± 36	0.024	50 ± 32	41 ± 49	0.236

### Hemodynamic parameters and recoil failure

**Figure 4:**
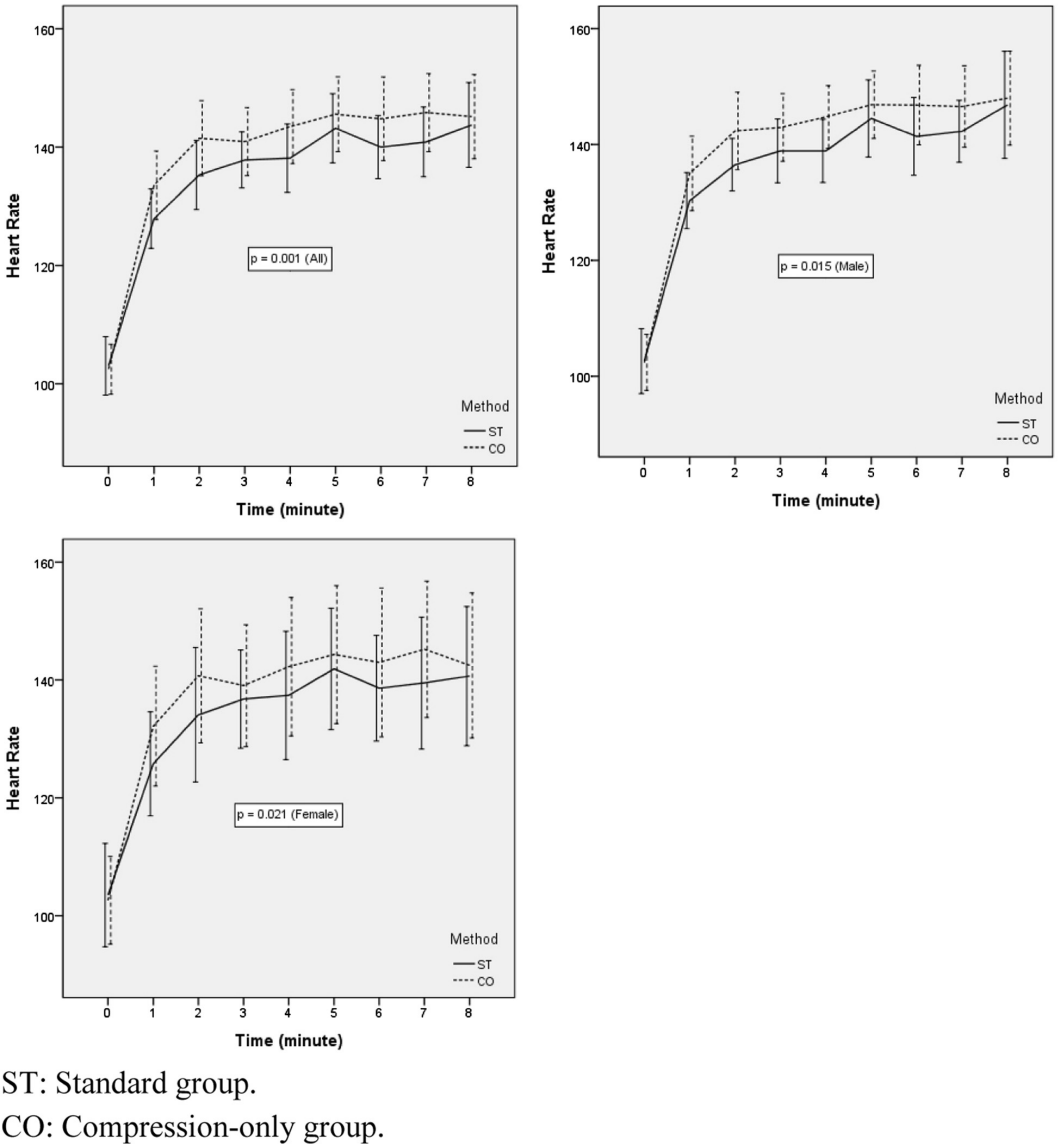
**The change in heart rate with each CPR method.**

The change in the mean blood pressure before and after each CPR was not significantly different between the 2 types of CPR (p =0.980). However, the change in the heart rate before the start of CPR to the end of CPR was significantly different between each type of CPR (p =0.001) (Figure 4). When the analysis was performed on the basis of gender, the change in the heart rate was significantly different in the male and female participant groups between the 2 CPR methods (p =0.015, p =0.021, respectively). The change in the heart rate was also statistically significant during CPR (p =0.007). The rate of recoil failure during the total 8 minutes was not significantly different between the 2 types of CPR (p =0.958) (Table 4).

**Table 4:** **The percentage of recoil failure after chest compression and the hands-off time during 8 minutes**

	**ST-CPR (mean ± SD)**	**CO-CPR (mean ± SD)**	**p-value**
Recoil failure after chest compression (%)	2.2 ± 12.1	2.4 ± 9.4	0.958
Hands-off time for each ventilation pause (s)	3.9 ± 4.1	0	.

## Discussion

In this study, we found that the rate of adequate CCs was higher in the ST-CPR group than in the CO-CPR group after 2 minutes and the number of adequate CCs was higher in the ST-CPR group than in the CO-CPR group during the total 8 minutes of CPR. The heart rate was increased more in the CO-CPR group than in the ST-CPR group during the total 8 minutes.

The significance of chest compression compared with ventilation and drug administration during CPR has been emphasized over early defibrillation. The recommended rate and depth of CC have increased (push hard and push fast) according to the updated CPR guidelines. The reason for this is that effective chest compression is essential to provide blood flow during CPR [5-7,13,14]. Healthcare providers and laypersons have shown reluctance to perform mouth-to-mouth ventilation for victims of cardiac arrest, and this remains a theoretical and potential barrier to performing bystander CPR [15-18]. The simpler chest compression-only technique may help overcome panic and hesitation to act. Therefore, CO-CPR is encouraged for untrained lay-rescuers in the 2010 CPR guidelines [19,20]. However, if the trained lay-rescuer or healthcare provider is able to perform rescue breaths between CC, he or she should add rescue breaths in a ratio of 30 CCs to 2 breaths and continue CPR until an automated external defibrillator arrives, emergency medical service (EMS) providers take over care of the victim, or an advanced airway is placed. There are many rescuers and feedback systems, and the CPR leader can co-ordinate rescuer change or control the overall CPR progress during real advanced life support [20,21]. However, in real situations, other rescuers and feedback devices are not always available; and only 1 bystander can perform CPR until the EMTs or healthcare providers arrive at the scene. With increasing urbanization, the time interval between the call and EMS arrival is also increasing; therefore, the lay-rescuer may have to perform longer CPR alone. Delay to the initiation of BLS and advanced life support intervention negatively affects the outcome from prehospital cardiac arrest [22]. In a nationwide population-based study of EMS-assessed out-of-hospital cardiac arrest and CPR surveillance data, the median time intervals between the call and EMS arrival and the call and hospital arrival were found to be 6 minutes (interquartile range, 5–9 minutes) and 22 minutes (interquartile range, 16–30 minutes), respectively [22,23]. Consequently, guidelines were modified to emphasize the depth and rate of CC and to recommend CO-CPR for untrained lay-rescuers and sole bystanders, potentially resulting in rescuer fatigue and affecting chest compression quality. Further studies comparing the outcomes of bystander CPR following the 2005 vs. 2010 CPR guidelines should be performed in the future.

In this study, male participants performed better than female participants in terms of rate and number of adequate chest compressions. In addition, the mean rate of adequate CCs per minute was maintained above 70% during the 8 minutes of ST-CPR by male participants, but it decreased to below 70% after only 2 minutes of CO-CPR. In contrast, the mean rate of adequate CCs per minute was decreased below 20% after 1 minute of ST-CPR and CO-CPR in female participants. Therefore, there was a clear difference in CPR performance between male and female participants. These results are in agreement with a previous simulation study performed in accordance with the 2005 guidelines, which showed a significant gender difference in the time-dependent change in mean proportion of correct CCs [24]. However, the gender difference in the chest compression quality was wider in our study following the 2010 guidelines than in the previous study following the 2005 guidelines. Therefore, when a female layperson is educated for bystander CPR, the instructor should consider this gender difference in CPR performance. In many studies, bystander CPR was found to influence the CPR outcome [25-28]. Therefore, the recent CPR guidelines should recommend bystander CPR for BLS with ST-CPR or CO-CPR. If the CPR performance quality of female bystanders is significantly lower than that of male bystanders in real situations, a difference in the CPR outcome may occur. Further studies assessing the effect of bystander’s gender on the CPR outcome should be performed.

In this study, during the 8-minute long experiment, the number of CCs during ST-CPR was higher than that during CO-CPR after 6 minutes with a 4 cm depth and after 2 minutes with a 5 cm depth. This means that ST-CPR may be useful during bystander CPR with a 5 cm depth of chest compression according to the 2010 CPR guidelines. Typically, only 1 rescuer is available to perform CPR unassisted on a patient with cardiac arrest before the arrival of EMT or healthcare providers; therefore, in this context, ST-CPR may be more useful in providing effective CC than CO-CPR in patients.

The heart rate of the participants, which may reflect CPR fatigue, was continuously measured in our study. The increase in the heart rate was higher with CO-CPR than with ST-CPR during 8 minutes of CPR. Rescuer fatigue may occur more quickly in cases of bystander CO-CPR compared to ST-CPR in the absence of another rescuer. Recent studies on CPR fatigue have generally used an objective fatigue scale, such as the visual analogue scale [29,30]. However, this objective method for measuring fatigue may be inaccurate owing to differences in individual thresholds of fatigue. Our measurement of heart rate was the first objective analysis of the effects of fatigue during 1 rescuer CPR according to the 2010 CPR guidelines.

In a previous study in a small number of CPR-trained bystanders performing CPR, the common reasons cited for not performing CPR were panic and inability to perform CPR correctly [31]. In addition, the previously reported reasons for not performing CPR (mouth-to-mouth, infectious-disease risk) were not the reasons which the bystanders cited for not performing CPR. In such a case, high quality CPR training for laypersons should be carried out, which emphasizes real simulations, motivation, and confidence development for CPR methods other than CO-CPR. According to the results of our 8-minute simulation study, rescuer fatigue can develop quickly and rescuer performance is lower in CO-CPR than in ST-CPR. Therefore, CPR educators and learners should remember these points, and a further study is needed for determining which CPR method is more effective during the dispatcher-assisted CPR according to the field situation and the degree of CPR education of laypersons.

### Limitations

This study has some limitations. Firstly, this is a manikin study; therefore, the adequate depth of CC may be different from that in CPR for a real cardiac arrest patient in consideration of chest stiffness and damping [32]. In addition, the participant’s attitude towards manikin CPR may be different from that towards actual CPR. Secondly, the participants in this study were younger than the average bystander. In addition, participants in this study belonged to a homogeneous age group, but the average bystander is heterogeneous in terms of age. A further study including various human groups is needed. Thirdly, the time period of bystander CPR before EMT arrival may be longer than 8 minutes. Because we only investigated the changes over 8 minutes, we cannot conclude about the differences in quality between ST-CPR and CO-CPR on the basis of 2010 CPR guidelines after more than 8 minutes of CPR.

## Conclusion

The rate of adequate chest compressions per minute was lower with CO-CPR than with ST-CPR during 8 minutes of CPR. Specifically, although the number of adequate chest compressions was higher with CO-CPR than with ST-CPR during the first 2 minutes, it was higher with ST-CPR than with CO-CPR after 3 minutes. The increase in the heart rate was higher with CO-CPR than with ST-CPR during 8 minutes of CPR. The decreasing rate of adequate CCs per minute between ST-CPR and CO-CPR was significant in male participants. In contrast, women did not adequately perform CC from the start of CPR with either of the CPR methods.
